# Wastewater-Based Epidemiological Surveillance in France: The SUM’EAU Network

**DOI:** 10.3390/microorganisms13020281

**Published:** 2025-01-26

**Authors:** Frédéric Jourdain, Laila Toro, Zoé Senta-Loÿs, Marilyne Deryene, Walid Mokni, Tess Azevedo Da Graça, Yann Le Strat, Sofiane Rahali, Ami Yamada, Anna Maisa, Maël Pretet, Jeanne Sudour, Christophe Cordevant, Thierry Chesnot, Veronica Roman, Amandine Wilhelm, Benoît Gassilloud, Damien Mouly

**Affiliations:** 1 Occitanie Regional Office, Regional Division, Santé Publique France (French National Public Health Agency), 31050 Toulouse, France; damien.mouly@santepubliquefrance.fr; 2 General Directorate for Health, Ministry of Health, 75007 Paris, Francewalid.mokni@sante.gouv.fr (W.M.); 3 Data Division, Santé Publique France (French National Public Health Agency), 94415 Saint-Maurice, France; 4 Regional Division, Santé Publique France (French National Public Health Agency), 94415 Saint-Maurice, France; ami.yamada@santepubliquefrance.fr; 5 Infectious Diseases Division, Santé Publique France (French National Public Health Agency), 94415 Saint-Maurice, France; 6 Strategy and Programs Department, Research and Reference Division, ANSES, 94701 Maisons-Alfort, France; christophe.cordevant@anses.fr; 7 Nancy Laboratory for Hydrology, ANSES, 54000 Nancy, Franceveronica.roman@anses.fr (V.R.);

**Keywords:** wastewater, surveillance, epidemiology, France, public health, SUM’EAU, SARS-CoV-2, COVID-19

## Abstract

Wastewater surveillance is a powerful public health tool which gained global prominence during the COVID-19 pandemic. This article describes the development and implementation of the national wastewater surveillance network in France: SUM’EAU. Preliminary work included defining a sampling strategy, evaluating/optimising analytical methods, launching a call for tenders to select network laboratories and producing wastewater monitoring indicators. SUM’EAU was then deployed in three stages: (i) a pilot study, (ii) the transfer of analytical activities from the National Reference Laboratory to four selected network laboratories, and (iii) the extension of the system to additional sampling sites. Currently, SUM’EAU monitors SARS-CoV-2 across 54 wastewater treatment plants in mainland France. Once a week on business days, 24 h flow-proportional composite samples are collected at plant inlets and transported at 5 °C (±3 °C) to partner laboratories for analysis. The analytical process involves sample concentration, RNA extraction, and digital RT-PCR/q-RT-PCR to detect and quantify the presence of the SARS-CoV-2 genome in wastewater. Subsequently, data are transferred to *Santé publique France*, the French National Public Health Agency, for analysis and interpretation. While SUM’EAU has been instrumental in monitoring the COVID-19 pandemic and holds significant potential for broader application, securing sustainable funding for its operation remains a major challenge.

## 1. Introduction

Wastewater surveillance (WWS) has served as a valuable public health tool for decades, particularly within the Global Polio Eradication Initiative (GPEI) (https://polioeradication.org/, accessed on 24 January 2024). However, the COVID-19 pandemic increased global attention to wastewater-based epidemiology (WBE), demonstrating its value as an innovative, cost-effective, and adaptable approach to monitor disease spread across populations [1,2,3]. Unlike traditional methods that rely on clinical testing or surveys, WWS offers a comprehensive snapshot of community health by analysing contaminants present in sewage, thereby reflecting broader health trends [4,5]. It operates on a simple principle: substances excreted in urine/faeces often leave traces in wastewater. These include a wide array of indicators such as viruses, licit and illicit drugs, and dietary biomarkers. Through wastewater analysis, public health authorities can gain insights into population health and lifestyle trends and better understand patterns of disease transmission [1,4,6].

This article describes the development and implementation of the national WWS system in France: SUM’EAU (“*Surveillance microbiologique des eaux usées*” or “microbiological wastewater surveillance”). Established under the supervision of the Ministry of Health, SUM’EAU was created with technical support from two governmental agencies: the French National Public Health Agency, *Santé publique France* (*SpF*), responsible for epidemiological surveillance, and the French Agency for Food, Environmental and Occupational Health & Safety (ANSES), which provides laboratory expertise as the National Reference Laboratory (NRL) for SARS-CoV-2 in wastewater and sewage sludge. The network was designed to complement existing population-based surveillance systems and inform public health decision-making. Currently, SUM’EAU focuses on monitoring severe acute respiratory syndrome coronavirus 2 (SARS-CoV-2) in France. Its creation aimed to institutionalise and capitalise on national [7,8,9,10] and international WWS initiatives that emerged during the COVID-19 pandemic, while adhering to guidelines set by the European Commission [11] and World Health Organization (WHO) [4].

## 2. Materials and Methods

### 2.1. Wastewater Sampling Strategy

#### 2.1.1. Wastewater Surveillance Objectives

The WWS of SARS-CoV-2 operates like a sentinel surveillance system, providing rapid access to high-quality data from a limited number of sites, with a focus on monitoring trends [12]. SUM’EAU was designed with two main surveillance objectives: (i) the early detection of pathogen circulation in the population and (ii) the monitoring of trends. Its sampling strategy was developed to optimise resource allocation in line with these goals

#### 2.1.2. Spatial Resolution

Sampling at the inlet of wastewater treatment plants (WWTPs) is common practice for WBE due to its numerous advantages. It enables the collection of data representing the entire population served by a plant with a single sample, providing a broad and cost-effective measure of community health. Additionally, raw wastewater is more likely to accurately reflect viral circulation within a population, as it remains unaffected by the wastewater treatment processes [13]. To identify the most adapted WWTPs for sampling amongst France’s 20,000+ facilities, three main criteria were considered: (A) the size of the area; (B) the geographic coverage on the department level; and (C) the capacity of the WWTP.

The size criterion (A) considered the European Commission’s recommendation of 17 March 2021 (2021/472), which suggests that WWS systems should, at a minimum, include wastewater from large cities with populations exceeding 150,000 inhabitants [11]. To identify these areas, we analysed the French territory at the Urban Unit (UU) scale. A UU is defined as a commune or group of communes forming a continuous built-up area with no gaps larger than 200 meters between buildings, and with at least 2000 inhabitants [14]. As of 2017, 47 UUs in France exceed the threshold of 150,000 inhabitants, representing over 475 WWTPs, 29.3 million people and covering 45 of the 101 French departments [11]. To ensure representativeness, it was decided that at least one WWTP would be selected from each of the 45 French departments (criterion B). For the 56 French departments that did not have UUs of at least 150,000 inhabitants, the largest WWTP within the largest UU in each was included.

To assess criterion C, the capacity of wastewater facilities was assessed based on their population equivalent (PE), where one PE represents the organic biodegradable load of a five-day biochemical oxygen demand of 60 g of oxygen per day [15]. A threshold of 100,000 PE was set, as it aligns with the requirement for more frequent influent monitoring under French urban wastewater treatment regulations [16]. Of the 47 UUs with populations exceeding 150,000, 43 contain 76 WWTPs with nominal capacities greater than this threshold. For the remaining four UUs, the largest WWTP in each of these was selected instead.

**Figure 1:**
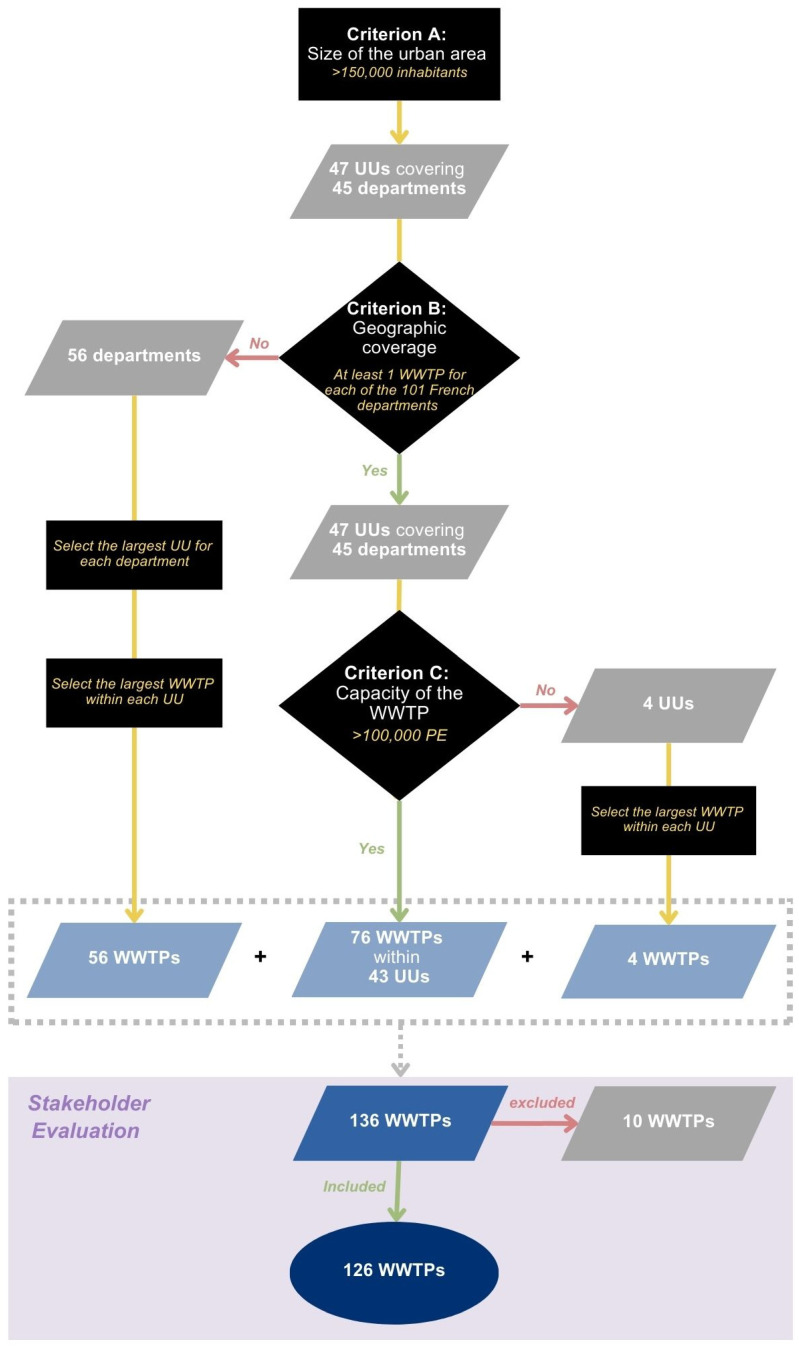
Selection process for sampling sites based on the size of the area (criterion A), geographic coverage on the departmental scale (criterion B), and the capacity of the WWTP (criterion C). UU: Urban Unit; WWTP: wastewater treatment plant; kg: kilogram; PE: population equivalent.

**Figure 2:**
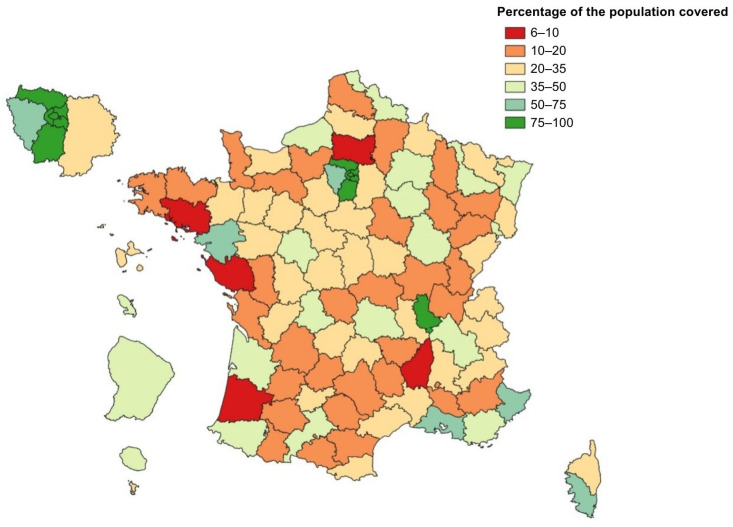
Percentage of the population covered per department by the 126 WWTPs selected for the SUM’EAU network.

These criteria allowed for the identification of a preliminary list of 136 WWTPs, which was presented to various stakeholders along with a detailed report on the selection process. Participants included representatives from public health authorities; the Ministry of Ecology, responsible for wastewater infrastructure compliance; and professionals from the water and sanitation sector, including WWTP operators and academics at the forefront of wastewater surveillance in France at the time. Through structured discussions and feedback sessions, stakeholders evaluated WWTPs based on (i) operational and logistical feasibility, (ii) balanced geographic representation to avoid over-representation of certain metropolitan areas (e.g., the Greater Paris area), and (iii) operational quality, ensuring the exclusion of plants with known deficiencies (e.g., parasitic water infiltration) that could compromise analysis accuracy and result interpretation. Ultimately, the list was refined to 126 WWTPs of interest (Figure 1): 70 located in UUs with more than 150,000 inhabitants, and 56 in UUs with fewer than 150,000 inhabitants. At least one sampling point was retained per department, providing a cumulative coverage of approximately 47% of the French population. The variation in coverage rates across them reflects geographic disparities in the country, with some areas being significantly more rural than others (Figure 2). Coverage was deemed satisfactory, especially given that nearly 20% of the population is not connected to a collective sanitation system.

#### 2.1.3. Wastewater Sampling Frequency

Wastewater flow rates experience both inter- and intra-day variations due to factors such as precipitation, industrial discharges, and domestic usage, which can affect the composition of wastewater both qualitatively and quantitatively. Hence, to favour sample representativeness, composite sampling is typically preferred. This method involves collecting samples at predetermined intervals, with fixed or flow-proportional volumes, typically over a 24-h period [17,18,19,20,21,22]. In France, 24-h flow-proportional composite sampling is performed routinely at the inlet of all WWTPs with nominal capacities exceeding 600 kg of biochemical oxygen demand (BOD) per day [16]. This approach also aligns with the European Commission’s 2021/472 recommendations for sampling and was therefore selected as the sampling method for SUM’EAU [11]. Scientific evidence at the time of evaluation (October 2022) suggested sampling two to three times per week to be ideal, balancing cost and performance, with non-consecutive samples being favoured [23,24,25]. The European Commission’s 2021/472 recommendations for the systematic surveillance of SARS-CoV-2 and its variants similarly advise sampling twice per week [11]. However, the optimal sampling frequency also depends on WWS objectives and the intensity of pathogen circulation. More frequent sampling may be warranted in situations with low pathogen circulation to enhance the resolution of detection and trend monitoring [26,27,28]. Accordingly, intensive sampling across multiple sites may not be justified from a cost–benefit perspective, given its limited added value for public health decision-making. Sampling frequency should be adapted to the local epidemiological context and risk management strategies. For the routine surveillance of SARS-CoV-2 in France, weekly sampling was deemed sufficient. This strategy aligns with the WHO’s 2022 and later 2023 recommendations for the environmental surveillance of SARS-CoV-2, which consider weekly sampling appropriate for the long-term monitoring of viral circulation [4,29]. Furthermore, this sampling frequency is consistent with weekly epidemiological analyses of multi-source human case data, including syndromic, virological, and genomic surveillance data, conducted by SpF.

### 2.2. SUM’EAU Implementation: A Multistep Process

**Figure 3:**
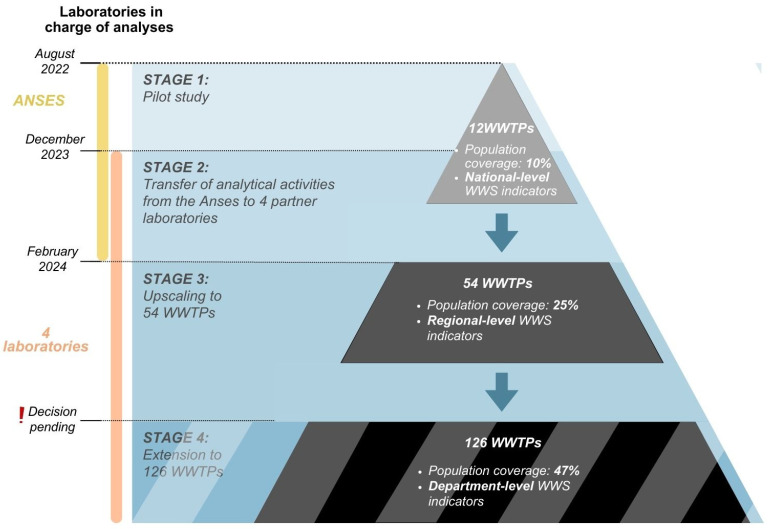
SUM’EAU implementation stages. WWTP: wastewater treatment plant; WWS: wastewater surveillance.

In September 2021, ANSES’ Nancy Laboratory for Hydrology was designated by the ministries in charge of health and the environment as the NRL for monitoring SARS-CoV-2 in wastewater and sewage sludge [30]. As NRL, it is responsible for harmonising virus detection methods and supporting SUM’EAU partner laboratories by assessing the efficiency of their detection systems through interlaboratory proficiency testing and evaluations. The implementation of the current SUM’EAU network was carried out in three stages: (i) a pilot study involving 12 WWTPs with wastewater analyses performed by the NRL, (ii) the transfer of analytical activities from the NRL to four partner laboratories, (iii) extension to 54 WWTPs in February 2024 (Figure 3).

#### 2.2.1. Pilot Study on 12 WWTP

In August 2022, a pilot study was launched on a subset of WWTPs to allow the NRL to evaluate and compare different analytical methods and optimise the sample preparation step. The aim was to establish a benchmark method for SARS-CoV-2 analysis for the SUM’EAU network. One plant was chosen from each region of metropolitan France, excluding Corsica. Selection also considered representation of the country’s diverse climate zones and prioritised urban areas served by a single WWTP to facilitate comparisons with epidemiological indicators. Of the 126 WWTPs identified, 12 were retained in the urban areas of Dijon, Grenoble, Lille, Marseille, Nancy, Nantes, Orléans, Paris, Pau, Rennes, Rouen, and Toulouse. These cover approximately 10% of the French population and provide national-level indicators of SARS-CoV-2 circulation in wastewater. All molecular analyses were performed by ANSES.

#### 2.2.2. Operational Transfer and Network Expansion

To facilitate the extension of the SUM’EAU network, the French Directorate General for Health launched a call for tenders for the selection of laboratories that would take over routine analyses. To organise the public market, the French territory was divided into different geographic lots based on logistical considerations, and interested laboratories were invited to submit bids on a lot-by-lot basis. Subsequently, submissions were evaluated based on two weighted criteria: the technical quality of the proposal (60% of the total score) and the cost of services (40% of the total score).

The technical quality assessment for each laboratory focused on several key factors, including their (i) organisation of services, available resources (human and equipment), and capacity to extend analyses to additional WWS targets of interest; (ii) analytical performances; (iii) management quality and certified accreditations; and (iv) internal quality control robustness. This approach ensured that selected facilities met high technical and operational standards.

Ten laboratories bid for one or more lots, of which four were selected. The awarded lots collectively cover the entire French metropolitan area. However, no bids were accepted for overseas territories due to either a lack of submissions or the failure of those received to satisfy the technical quality criterion. The NRL oversaw the transfer of analytical activities from the initial 12 WWTPs to the laboratories selected.

The network was then scaled up to an intermediate stage enabling regional-level WWS indicators to be generated. Selection aimed to include at least three WWTPs per region of metropolitan France (except for Corsica, which only has one), maximising spatial and population representation, while avoiding the over-representation of the most densely populated regions. As of February 2024, 54 WWTPs are monitored under SUM’EAU, covering approximately 25% of the French population. A second extension of the network to 126 WWTP, which would increase the resolution of WWS indicators to the departmental level, is currently under discussion. Of note, while the four SUM’EAU partner laboratories run routine wastewater analyses in their respective lots, the NRL continues to monitor the 12 original WWTPs on a weekly basis to validate data quality and comparability. It also operates an aquatech biobank through which it stores several fractions and extracts of weekly wastewater samples at −20 °C (±5 °C) and −75 °C (±5 °C), respectively. The aquatech serves as a backup system to allow potential result confirmation or retrospective investigation on parameters of interest.

### 2.3. Information Acquisition and Processing Workflow

SUM’EAU’s workflow for information acquisition and processing, from sampling to the interpretation of weekly wastewater data, involves four main stakeholders: (i) WWTP operators, charged with collecting and preparing samples for transport; (ii) the four SUM’EAU network laboratories, which process samples and conduct microbiological analyses; (iii) ANSES Nancy laboratory for hydrology, responsible for ensuring the reliability of methods and quality of analytical results on samples from the 12 original WWTPs; and (iv) SpF, which manages the quality control and statistical processing of the data, as well as the production, interpretation and communication of wastewater indicators.

### 2.4. Sample Concentration, Extraction, and Quantification

Once a week, 24 h flow-proportional composite samples of at least 200 mL are collected at the inlets of WWTPs and transferred to network laboratories and the NRL for analysis. The transfer occurs within 24 h of the end of sampling, with samples maintained at a controlled temperature of 5 °C, with a tolerance range of ± 3 °C. It should be noted that for the routine surveillance of SARS-CoV-2 in France, sampling and analyses are performed exclusively on wastewater. Upon reception, laboratories validate sample integrity by verifying the absence of leaks or bottle breakage (e.g., Diagnobag with absorbent) and are required to begin wastewater analyses within 24 h.

The analytical process includes sample concentration, RNA extraction, and RT-PCR to detect and quantify the presence of the SARS-CoV-2 genome in wastewater. Methods differ slightly between laboratories. For the concentration step, while laboratories 1 and 4 use an ultracentrifugation process on 11 mL samples, laboratory 2 uses a capture method on 40 mL samples, laboratory 3 an ultrafiltration method on 15 mL samples, and the NRL applies a precipitation method on 5 mL samples. Genome extraction is carried out using commercial methods, with volumes ranging from 200 to 500 μL depending on the laboratory. For molecular quantification, laboratories 1, 3, and 4 and ANSES use digital RT-PCR whereas laboratory 2 uses a qRT-PCR system. All laboratories apply a dual quantification approach targeting the E gene along with an additional target of their choosing. Laboratories 1, 3, and 4 use RdRp (IP4) as a second primer, while laboratory 2 and the NRL rely on the N1 gene. The ratio between the concentration of the E gene and the secondary target is systematically calculated, with significant deviations between the two indicating potential issues and the need to repeat analyses.

To ensure data quality, various control measures are used. All laboratories are required to include process-positive and process-negative controls and to apply these consistently during digital RT-PCR/q-RT-PCR. They must also actively monitor and document possible inhibition during analyses. Methods must meet specific performance criteria, including a limit of detection ≤ 1.10^4^ genome units of SARS-CoV-2 per litre (GU/L), and a limit of quantification ≤ 5.10^4^ GU/L. Finally, data quality and comparability across the four laboratories are verified weekly by comparing the ratio of E gene concentrations measured by the NRL for the 12 historic WWTPs to those measured by the network laboratories. Sustained deviations from ANSES data trigger an internal evaluation of methods to identify potential sources of non-conformity.

### 2.5. Wastewater Monitoring Indicators

#### 2.5.1. Normalisation Parameters

Monitoring pathogen circulation trends in wastewater requires normalising viral concentrations based on population size to account for dilution effects (from precipitation, industrial discharges, and domestic usage) as well as population movements and variations. This step is essential for providing context and improving the reliability and comparability of wastewater data over time and across sampling sites [31,32,33]. The European Commission recommends using wastewater flow rates for normalisation, alongside additional controls such as the crAssphage virus or pepper mild mottle virus (PMMoV) (2021/472) [11]. Other population normalisation factors are also proposed in the literature and include chemical oxygen demand (COD), biological oxygen demand (BOD5), ammonium (NH_4_-N), total nitrogen, total phosphorus, and Bacteroides HF183 markers [8,32,33,34,35,36,37,38,39]. Initially, the SUM’EAU network implemented a dual normalisation approach, utilising both wastewater flow rate and ammonium (NH_4_-N), an indirect marker of urine produced by urea hydrolysis.

Ammonium quantification offers several advantages: it (i) is supported by a standardised analytical framework; (ii) is less influenced by non-human sources; and (iii) enables dynamic (de facto) population estimates, better accounting for population movements in and out of the catchment areas [34,37,38,40]. Based on our experience, NH_4_-N is also more readily available, as it is analysed concurrently with SARS-CoV-2 detection. In contrast, flow rate data can be challenging to collect from WWTP. Moreover, they enable only fixed (de jure) population estimates, failing to account for fluctuations due to factors such as national holidays or weekday–weekend differences. Although wastewater flow rate data are still routinely collected, NH_4_-N has become the preferred method to produce normalised WWS indicators for routine SARS-CoV-2 surveillance.

#### 2.5.2. Data Processing

New WWS data are added weekly to an SQLite database and structured into several tables, including an indicator table used for data analysis. These data are then processed with R (version R-4.1.3), with outputs presented in HTML files rendered from Rmarkdown. This format facilitates interactive exploration and allows for a better understanding of the generated graphics. Subsequently, the data are averaged based on various geographical scales for visualisation and smoothed using the LOESS (Locally Estimated Scatterplot Smoothing) method to produce indicators. LOESS fits multiple local regressions around each data point using a subset of nearby points, enabling the identification of trends within datasets.

#### 2.5.3. Wastewater Monitoring Indicator Construction and Data Visualisation

**Figure 4:**
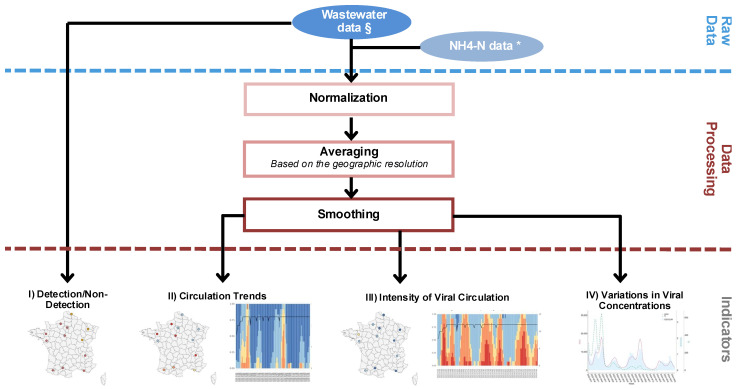
Summary of SUM’EAU’s wastewater indicators production process. * NH_4_-N: ammonium expressed in mg/L of ammonium (NH_4_) as nitrogen (N). § Genome concentration of SARS-CoV-2 determined using the E gene target for quantification and a second target (N1, N2, or IP4) for confirmation.

Four indicators were developed to visualise and interpret weekly SARS-CoV-2 wastewater data with regard to (I) detection/non-detection, (II) circulation trends, (III) intensity of viral circulation, and (IV) variations in viral concentrations (Figure 4). These can be applied across different geographic scales, including the national, regional, departmental and individual sampling site (WWTP) levels.

Indicator I qualitatively classifies wastewater results into three groups: (i) ‘Quantified’ for values exceeding the quantification limit; (ii) ‘Detected’, for values between the detection and quantification limits; and (iii) ‘Non-Detected, for values below the detection limit. Indicator II describes normalised SARS-CoV-2 circulation trends compared to two weeks prior, categorising trends into five levels defined arbitrarily: strong decrease (<−70%); decrease (between −70% and −10%); stabilisation (between −10% and 10%); increase (between 10 and 70%); and strong increase (>70%). Indicator III evaluates the intensity of viral circulation in relation to historical wastewater data, relativising weekly data against previous epidemic peaks and providing context to indicator II. For instance, a sharp rise in circulation trends (indicator II) does not necessarily equate to high viral circulation levels overall. Likewise, a decline in circulation does not always indicate low viral levels. Finally, indicator (IV) illustrates the variation in the ratio of SARS-CoV-2 concentrations to ammonium over time.

#### 2.5.4. Population-Based Reference Data

Several sources of population-based health data were used for comparison with normalised SARS-CoV-2 concentrations in wastewater (wastewater indicator or WWI) throughout the development of SUM’EAU. These included (i) the percentage of consultation attributed to suspected cases of COVID-19 in emergency departments from the OSCOUR^®^ network, which collects data from 93.3% of emergency visits nationwide [41], and (ii) COVID-19 incidence rates (Ti) per 100,000 inhabitants, derived from the number of RT-PCR and antigen tests performed and reimbursed by the French national healthcare system from the Information System for Population Screening (SI-DEP) diagnostic screening database [42]. To facilitate analyses, health data were aggregated by week at the department level corresponding to each wastewater treatment plant.

To evaluate the relationship between wastewater and epidemiological data, a correlation analysis was performed using Spearman’s rank correlation test. Separate evaluations were conducted for OSCOUR^®^ and incidence rates (Ti) against WWIs. Analyses used data from the 12 historic WWTPs up to 10 November and 9 June 2024, respectively. While diagnostic tests were fully reimbursed until 1 March 2023, only partial reimbursements are now provided [43], leading to a decline in the number of reported cases. To account for possible effects of policy changes related to COVID-19 diagnostic testing reimbursement, Spearman correlations were also performed for the periods before and after 6 March.

### 2.6. Communication of Wastewater Indicators

Since October 2023, SUM’EAU indicators have been published weekly in an open access governmental database https://www.data.gouv.fr, accessed on 24 January 2024 and incorporated into weekly epidemiological reports alongside other population-based surveillance data. This reporting helps to inform both the public and policymakers about epidemiological trends regarding transmissible respiratory diseases, supporting the implementation of preventive measures. As of October 2024, SUM’EAU indicators are also made available at the regional level.

## 3. Results

### 3.1. Operational Transfer of Routine Laboratory Analyses

**Figure 5:**
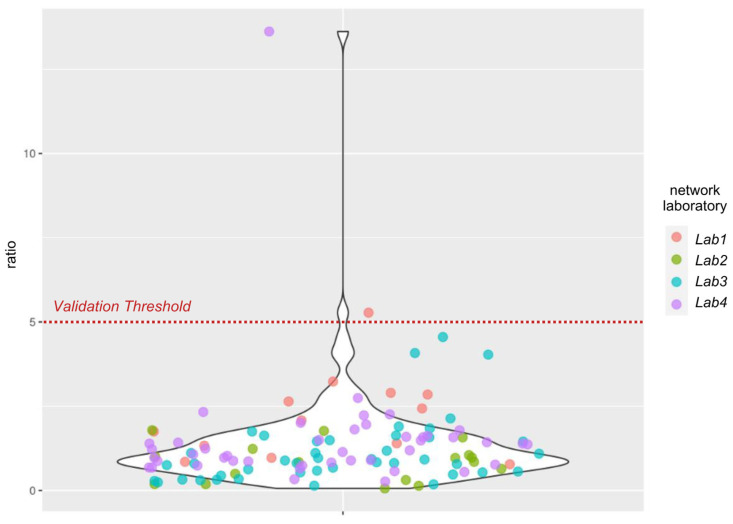
Ratio of E gene concentrations measured by the National Reference Laboratory to those measured by the four network laboratories.

The ratio of E gene concentrations measured by the NRL to those measured by the network laboratories was monitored closely over a 13-week period. This process enabled the validation of analytical methods for three laboratories, while corrective measures were implemented for the fourth. In the final follow-up, over 90% of results met the established threshold of five (Figure 5), allowing for the transfer of routine analyses to these laboratories. This indicator continues to be used to confirm the quality and comparability of WWS data from the four network laboratories.

### 3.2. SARS-CoV-2 Concentrations in Wastewater

**Figure 6:**
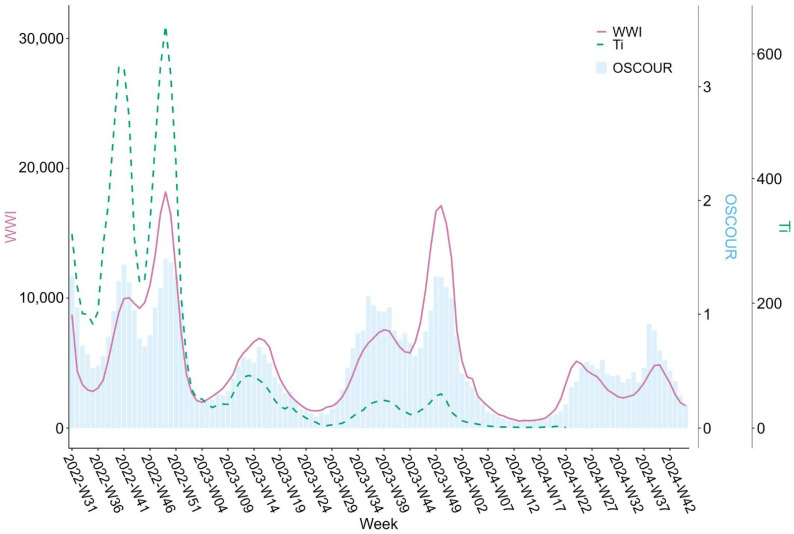
Comparison between three sources of epidemiological data: (i) normalised SARS-CoV-2 concentrations in wastewater (WWI) (represented by the pink line), (ii) COVID-19 incidence rates (Ti) (represented by the green dashed line), and (iii) OSCOUR^®^ indicators, for 12 wastewater treatment plants (represented by the blue histograms). WWTP: wastewater treatment plant; WWI: wastewater indicator—ratio of viral concentration of SARS-CoV-2 to concentration of ammonium nitrogen; Ti: incidence rate per 100,000 inhabitants; OSCOUR^®^: number of emergency room visits associated with suspected COVID-19 per 100 visits; W: week.

Normalised SARS-CoV-2 concentrations in wastewater (WWI) in genome units of SARS-CoV-2 per milligrams of NH_4_-N for the 12 historic WWTPs, COVID-19 incidence rates (Ti), and COVID-19-related consultations from emergency departments nationwide (OSCOUR^®^) were compared (Figure 6). The time series showed seven peaks in circulation between August 2022 and November 2024, which followed similar trends as OSCOUR^®^ data. The highest peaks in both WWI and OSCOUR^®^ data occurred in November and December of 2022 and 2023, with levels approaching 18,000, while the lowest levels ranged between 1000 and 3000. The average WWI value for the monitoring period (25 July 2022 to 10 November 2024) was roughly equal to 5000.

WWI values started to rise between one to two weeks before COVID-19-related emergency room visits, with longer delays observed during the May-June 2024 wave. The ratio between the WWI and OSCOUR^®^ data varied across waves. There was also strong consistency between the WWI and the Ti data trends, although this relationship decreased towards the end of 2022.

### 3.3. Correlation Between SUM’EAU and Epidemiological Data

**Figure 7:**
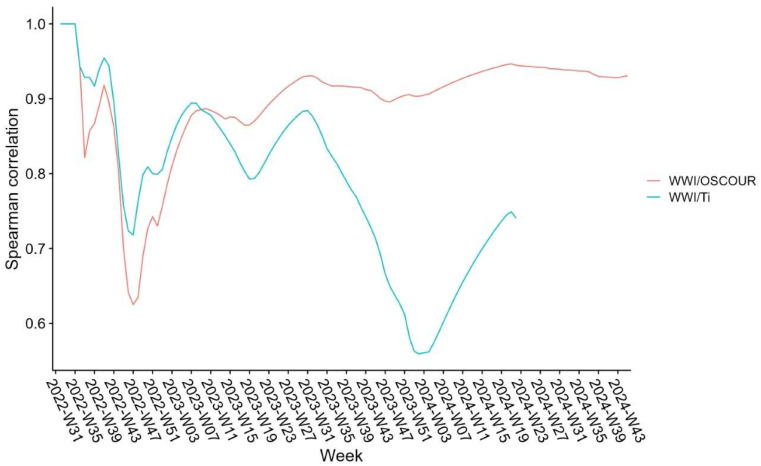
Correlations between the wastewater indicator and two epidemiological indicators: incidence rate (Ti) and OSCOUR^®^ emergency room visits attributed to SARS-CoV-2 rate per 100,000 people. WWI: wastewater indicator—ratio of viral concentration of SARS-CoV-2 to concentration of ammonium nitrogen; OSCOUR^®^: proportion of emergency room visits associated with COVID-19 per 100 visits; Ti: incidence rate per 100,000 inhabitants; W: week.

The Spearman correlation revealed a strong overall relationship between the SUM’EAU WWI and OSCOUR^®^ data (ρ = 0.93; *p*-value < 2.2 × 10^−16^) as well as between WWI and Ti (ρ = 0.73; *p*-value < 2.2 × 10^−16^). While the WWI/OSCOUR^®^ correlation remained stable over time, the correlation between WWI and Ti was more variable (Figure 7).

When analysed over two distinct periods—from the end of July 2022 to 6 March 2023 (period 1), and from 6 March 2023 to 10 November 2024 (period 2)—the WWI/Ti correlation was improved for period 1 (ρ = 0.89; *p*-value = 2.4 × 10^−7^). Indeed, while these indicators produced similar results with WWI/OSCOUR^®^ prior to March 2023, the WWI/Ti values diminished thereafter (period 2: ρ = 0.81; *p*-value = 2.2 × 10^−16^).

## 4. Discussion

The SUM’EAU WWS network has been operational since August 2022. Now including 54 WWTPs across mainland France, it samples wastewater once a week on business days and provides routine population-level data on SARS-CoV-2 circulation. The initial direction for the development of SUM’EAU anticipated a second system scale-up to 126 WWTPs. Nevertheless, to ensure the network’s long-term sustainability, its priorities need to be reconsidered and refocused going forward. Increasing the number of WWTPs to 126 would expand population coverage and allow the production of department-level data. However, this would also require significant investments in time, money, and resources, as well as complex logistical efforts, where producing department-level indicators may no longer be the top priority.

An alternative path forward could involve a more moderate increase in the number of WWTPs monitored within the SUM’EAU network to improve the robustness of regional indicators, especially in regions that are currently monitoring only three plants. There is also strong interest in expanding the network to include French overseas territories. However, challenges remain due to the lack of laboratories identified in the call for tenders that meet the technical quality criteria for selection, and resulting logistical constraints if samples need to be transported to metropolitan France for analysis.

Extending WWS to other targets has also been considered. By remodelling the system to use multiplex PCR methods capable of detecting multiple pathogens from RNA or DNA extracts, the network’s efficiency and scope would be significantly enhanced [44]. This approach could provide valuable public health data, while increasing the network’s flexibility, enabling SUM’EAU to better address both planned needs (e.g., regulatory requirements) and emerging public health threats. Under the revised EU urban wastewater treatment directive, member states will be required to expand wastewater monitoring beyond the SARS-CoV-2 virus and its variants to include other pathogens such as poliovirus, influenza viruses, antibiotic resistance, and other emerging threats [45]. In addition, an adaptable WWS system that can pivot more easily to other targets would increase France’s public health preparedness.

Integrating additional pathogen targets could be achieved at a lesser cost. Currently within SUM’EAU, sample transport, concentration, and preparation for screening account for roughly 35% of the total costs associated with WWS. Multiplex PCR could help offset these expenses, as these steps could be performed once for multiple pathogens. Needs related to sampling frequency and locations may also differ depending on the pathogen monitored. For example, in a polio-free country such as France, the GPEI recommends monthly sampling [5]. In such a scenario, it may also be warranted to focus surveillance efforts on a subset of WWTPs in higher-risk areas. Nonetheless, identifying relevant and adapted WWS targets requires careful consideration and may involve evaluating potential pathogens through a structured framework, as was performed to identify pathogens that could be priority targets for WWS during the 2024 Paris Olympic and Paralympic Games [46]. Incorporating additional pathogens into the SUM’EAU network would also require close collaboration with the research community, particularly to adapt analytical methods for emerging pathogens. Discussions about the direction and next steps in the evolution of the SUM’EAU network are ongoing.

WBE measures are inherently complex and prone to variability. Uncertainty can be introduced at various stages from sample collection to analysis, due to factors such as sampling conditions, the physico-chemical properties of samples, and potential interferences from molecules present in wastewater [47]. Combined with SUM’EAU’s focus on outcome-oriented performance objectives rather than process-driven objectives for its partner laboratories, this underscores the need for robust routine quality assurance measures. To ensure their proficiency in detecting and quantifying SARS-CoV-2 in raw wastewater samples, all network laboratories are required to participate in at least one annual interlaboratory evaluation and are encouraged to engage in additional trials organised by the NRL.

The COVID-19 pandemic has underscored the value of WWS as a non-invasive, cost-effective public health tool, capable of serving as an early warning system. By passively collecting community-level data, WWS ensures individual privacy, gathers information regardless of health status (symptomatic, asymptomatic) and reduces bias-related to clinical testing access and individual test-seeking behaviour [4,17]. For example, the differences in ratios between WWI and OSCOUR^®^ data observed across circulation peaks (Figure 6) likely reflect changes in care-seeking behaviours over the course of the pandemic, as well as differences in coding practices by emergency physicians, particularly during periods of co-circulation of multiple respiratory viruses. Moreover, while COVID-19 incidence rates reported through the SI-DEP system in France were influenced by national policy changes, due to its independence from clinical testing strategies and practices, SUM’EAU data remained unaffected (Figure 7). This further highlights the reliability and consistency of WWS. As of 27 May 2024 (week 22 of 2024), SARS-CoV-2 concentrations are compared only with OSCOUR^®^ data, Ti is no longer considered to be a representative indicator of the epidemiological situation in France.

WWS also has several limitations. As the tool provides aggregated data from all individuals within a defined catchment area, it cannot offer precise estimates of the number of infected persons contributing to a sample, nor can it link detection to an individual clinical case [29]. Likewise, the tool is unable to provide case-based data such as risk factors or severity of the disease. As such, WWS is used to complement other population-based surveillance systems, each of which has its own set of strengths and limitations.

Despite the heightened interest since 2020 in applying WWS to different targets and contexts, the effective use of WBE requires greater engagement and familiarisation among stakeholders, including decision-makers, epidemiologists, healthcare professionals, and the general public. Its integration has faced resistance, largely due to the traditional reliance on case-based data, which has fostered hesitancy regarding its use. As WWS is a relatively new tool in most settings, scepticism about the actionability of data persists [48]. Nevertheless, while management decisions in many cases do not rely solely on wastewater data, this does not diminish its value, particularly for the early detection of atypical pathogen circulation. To build trust in WWS, efforts should focus on raising awareness of this emerging data source, clearly communicating its strengths and weaknesses, and ensuring its better integration with other public health surveillance data (e.g., creating protocols to guide the use of WWS data for public health action). More case-based examples of the use of WWS data to support public health decision-making would also help to promote this tool.

## 5. Conclusions

WWS has been operational in France to help monitor the COVID-19 pandemic since August 2022. This tool has proven particularly useful for confirming trends seen across other data sources and guiding resource allocation decisions (staff, hospital beds, testing centres, vaccination centres). Nonetheless, as in many other countries in Europe, securing sustainable funding for WWS in France remains a major challenge.

The evolution of the EU regulatory landscape presents a unique opportunity to address these challenges. For instance, the revision of the urban wastewater treatment directive could serve as a catalyst to institutionalise WWS in the long term. Moreover, initiatives such as EU-WISH (https://www.eu-wish.eu/, accessed on 24 January 2024), a joint action aimed at strengthening WWS for public health throughout Europe, could play a pivotal role by addressing key challenges, including standardising practices, identifying priority WWS targets, and fostering knowledge exchange and best practice sharing. Discussions regarding the development of strategies for the sustainability of WWS in national policies are also ongoing as part of this project. Finally, the international collaboration led by the European Commission’s Joint Research Centre (https://wastewater-observatory.jrc.ec.europa.eu/, accessed on 24 January 2024) to advance the scientific and technical foundations of WWS, will help strengthen and maintain WBE’s role in public health surveillance and response going forward.

## Data Availability

Weekly SUM’EAU wastewater indicators are available as of 25 July 2022, on the open access platform of French public data: https://www.data.gouv.fr, accessed on 24 January 2024.
